# Early Sexual Debut and the Effects on Well-Being among South African Adolescent Girls and Young Women Aged 15 to 24 Years

**DOI:** 10.1080/19317611.2021.1979162

**Published:** 2021-10-28

**Authors:** Tracy McClinton Appollis, Kim Jonas, Roxanne Beauclair, Carl Lombard, Zoe Duby, Mireille Cheyip, Kealeboga Maruping, Janan Dietrich, Catherine Mathews

**Affiliations:** a Health Systems Research Unit, South African Medical Research Council, Cape Town, South Africa; b Department of Psychiatry and Mental Health, Division of Child & Adolescent Psychiatry, Adolescent Health Research Unit, University of Cape Town, Cape Town, South Africa; c South African DST-NRF Centre of Excellence in Epidemiological Modelling and Analysis (SACEMA), Stellenbosch University, Stellenbosch, South Africa; d Data Yarn, Pretoria, South Africa; e Centers for Disease Control and Prevention, Pretoria, South Africa; f Faculty of Health Sciences, Perinatal HIV Research Unit (PHRU), School of Clinical Medicine, University of the Witwatersrand, Johannesburg, South Africa

**Keywords:** Early sexual debut, HIV, adolescents, youth, South Africa

## Abstract

We compared first sex experiences and wellbeing of adolescent girls and young women (AGYW) who had an early sexual debut (age < 15) with those who had later sexual debut. We conducted a representative household survey among AGYW aged 15-24 years in six districts in South Africa. Of 3009 AGYW who had ever had sex, 8.9% reported early sexual debut. Early sexual debut was associated with coercion at first sex and a lower average well-being score compared with a later debut. Interventions which aim to delay early sexual debut may positively affect well-being.

## Introduction

Early sexual debut, defined as having sex for the first time under the age of 15 years (Richter et al., 2015), has been found to be associated with higher rates of subsequent condomless sex and multiple partnerships which can increase a young person’s risk for adverse sexual and reproductive health outcomes (e.g. sexually transmitted infections (STIs) and unintended pregnancies) (Kugler et al., 2017; Richter et al., 2015; Tsuyuki et al., 2019). The odds of becoming infected with HIV increases significantly among those who experience an early sexual debut (Anderson et al., 2007; Kibangou, 2015; Onsomu et al., 2013; Pettifor et al., 2004; 2005; Shrestha et al., 2016; Zuma et al., 2010).

Between 17.8 and 26.4% of male and 5.1 and 8.1% of female adolescents in South Africa report having had sex before the age of 15 years (Chirinda et al., 2012; Pettifor et al., 2009; Richter et al., 2015). This is of particular concern given South Africa has the largest HIV epidemic in the world, and adolescent girls and young women (AGYW) aged 15−24 years account for a quarter of new infections (Shisana et al., 2014). Gender inequalities, violence against women, and discrimination are barriers to AGYW’s ability to protect themselves from HIV (Joint United Nations Programme on, 2016). Physical intimate partner violence (IPV) has also been shown to be associated with inconsistent condom use in South Africa, placing AGYW at risk of HIV infection (Townsend et al., 2011). Furthermore, prevalence of physical or sexual IPV among women in sub-Saharan Africa is estimated to be over 30% (WHO, 2013). Additional South African studies show that AGYW who have had an early sexual debut are more likely to have been coerced into their first sexual experience (Chirinda et al., 2012; Mathews et al., 2009; Pettifor et al., 2009; Richter et al., 2015).

Early sexual debut, whether coerced or not, can also be associated with negative emotional experiences such as regret. “Regret” has been defined as a negative cognitive emotion, often accompanied by feelings of self-blame, disappointment with one self for action or inaction, and thoughts that the present would be different had one acted differently (Eshbaugh & Gute, 2008; Oswalt et al., 2005). Studies from Europe, North and South America, and Asia describe young people regretting their first sexual experience, or reporting sentiments of wishing they had waited longer to have their first sex (Cotton et al., 2004; Osorio et al., 2012; Wight et al., 2000). A study conducted in New Zealand among males and females, found that a substantial proportion of young women regret early intercourse (Dickson et al., 1998). However, to our knowledge, no studies have investigated regret after sexual debut in South Africa and the extent to which early sexual debut might impact well-being is unknown. The effects of regret on quality of life and well-being has however been researched in older participants (aged 19–35 years), showing that intense regret has a negative impact on well-being and quality of life (Wrosch et al., 2005).

One factor influencing sexual debut is age disparity between partners. Having a partner 6 or more years older (Kaestle et al., 2002) and 3 or more years older (Koon-Magnin et al., 2010) has been found to be associated with early sexual debut among American females. A Kenyan study found that AGYW aged 15-19 years had double the risk of HIV infection when their partner was 10 years older than themselves compared to those with partners 0 to 4 years older (Kelly et al., 2003). Studies also report that AGYW with older sexual partners are significantly less likely to report condom (Ford et al., 2001) and contraception use (Glei, 1999) which puts them at greater risk of HIV/STI transmission and unintended pregnancies possibly due to gender-power imbalances (Glei, 1999; Onsomu et al., 2013). Female condoms do offer AGYW a method to protect themselves against pregnancy and HIV/STI transmission, however, usage has been found to be low among AGYW aged 15-24 years for varying reasons such as the higher cost compared to male condoms, not being as available as male condoms, and difficulty in using them (Haffejee & Maharajh, 2019). Similarly, AGYW who report to have been coerced at first sex are more likely to report having had an STI and unintended pregnancy (Koenig et al., 2004; Maharaj & Munthree, 2007). Condom use at sexual debut has been shown to increase the likelihood of subsequent condom use (Shafii et al., 2004). In South Africa, HIV-negative participants who used condoms at first sex were 1.38 times more likely to remain uninfected than those who did not use condoms (Kincaid et al., 2014). Dual protection is critical in preventing both unintended pregnancy and HIV/STI infection among AGYW (Galarraga et al., 2018). Many factors influence condom use among young people. Qualitative research with South African AGYW and young men revealed negative beliefs and perceived negative side effects of condoms which served as a barrier to their use. For AGYW factors such as relationship security and the desire to demonstrate love, trust, intimacy and commitment, as well as the fear of violent reactions from male partners motivated the nonuse of condoms. For young men, increased sexual pleasure, proof of masculinity and power, gaining prestige amongst peers, desire to attain sexual prowess, respect, and masculine sexual maturity, all contributed in refusal to use condoms (Duby et al., 2021).

Despite evidence showing that adolescents (males and females) who have an early sexual debut are subsequently at higher risk of adverse sexual and reproductive health (SRH) outcomes (Kugler et al., 2017; Richter et al., 2015; Tsuyuki et al., 2019), little is known about whether AGYW are at greater risk of experiencing subsequent regret or disappointment about their first sex, and whether early sex has an adverse effect on well-being. In light of these unknowns, this study has three objectives: (1) describe AGYW’s first experience of penetrative sex by participants’ age at the time of the survey; (2) compare characteristics of these first sexual experiences among those who had an early sexual debut (under 15 years of age) and those who had a later sexual debut, stratified by age group; (3) investigate whether reporting an early sexual debut by AGYW had an adverse impact on well-being, adjusting for known confounders.

## Methods

### Study design

This study formed part of a cross-sectional household survey conducted as part of an evaluation of a combination HIV prevention intervention among AGYW aged 10–24, implemented 2016–2019 in ten South African districts. The intervention consisted of health, education and support services. A stratified cluster sampling design was used. The South African population was stratified into 10 districts within 7 provinces, then a random sample of small area layers (SALs) within each district was taken. Then households were sampled within SALs, and all AGYW living in sampled households between the ages of 15 and 24 years old were invited to participate in the household survey (For a more detailed description of the complete sampling strategy please see (Mathews et al., 2020)). The survey took place in six of the ten intervention districts from September 2017 to November 2018. A total 4399 AGYW aged 15–24 participated in the survey.

### Measures

*Demographic* information was obtained by asking questions around age, marital status, education and orphanhood status. *A binary indicator of socio-economic status (SES)* was created using a Cluster Analysis with the K-modes algorithm. Cluster Analysis is a machine learning technique that allows analysts to group data based upon shared features. The features used in the creation of this indicator were: (1) AGYW was away from home for more than one month in the past 12 months; (2) piped water in household; (3) flushing toilet(s) in household; (4) working electricity in household; (5) household has car; (6) household has computer; (7) household has access to internet; (8) household has refrigerator; (9) household has stove; (10) AGYW went a day/night without eating in past month; (11) AGYW has own money; (12) AGYW saves money; (13) AGYW owes money. These variables were grouped into clusters by the algorithm indicating those with a relatively low SES and those with a high SES.

Age at which *sexual debut* occurred was assessed by asking participants “The first time you had sex (vaginal/anal penetrative sex) – how old were you?”. Response options included: “younger than 10 years”, a list of ages between 10 and 19 years for participants to choose from, “older than 19 years”, and “Never had sex (Virgin)”. Early sexual debut was defined as sex before 15 years of age, and later sexual debut as having sex at 15 years of age or older. Sexual debut is an outcome in the analysis that compares characteristics of first sexual experiences, and also an explanatory variable in the analysis of whether early sexual debut affects subsequent well-being.

C*haracteristics of the participants’ first sexual partner* were determined by asking the participant who their first sex was with, and then providing the options: boyfriend (own age), boyfriend (+-5 years older), boyfriend (+10 years older), blesser (i.e. a relationship with an older male partner who typically provides pocket money), casual partner, stranger, father, husband, teacher, other (specify) and prefer not to say. The response options boyfriend (own age), boyfriend (+-5 years older) and husband then were grouped together to form one category, and referred to hereafter as “lower risk partners”, while the remaining options then grouped together to form a separate category referred to as “higher risk partners”. The reason for grouping first sexual partners in this manner is based on evidence that older partners, particularly those 10 or more years older, have double the risk of being HIV positive compared to partners 0 to 4 years older (Kelly et al., 2003). Furthermore, women with older partners are less likely to report condom use (Ford et al., 2001) and contraception use (Glei, 1999) possibly due to gender-power imbalances, and this puts them at greater risk of HIV/STIs transmission and unintended pregnancies.

*First sexual experience* was measured by asking participants whether their first sex was wanted, not wanted, forced on them, or was rape. Participants were considered *coerced* when participants answering yes to being “forced against their will” and “raped”.

*First Sex Condom Use* was assessed by asking “The first time you had sex – did you or the person you had sex with use a condom?”, and participants could answer “yes”, “no” and “prefer not to say”.

*Use of contraception at first sex* was measured by asking “The first time you had sex – did you use anything to prevent becoming pregnant?” The response options included a range of contraceptives; if AGYW answered yes to any *contraceptive methods other than condoms* (injection, implant, pill, intra-uterine device, diaphragm), they were classified as “modern contraceptive use”. To create a variable for *Dual Protection* responses from participants who indicated they used both a condom and another form of modern contraception were combined.

*Subsequent regret or disappointment about first sex* was measured by asking participants “Thinking about the first time you had sex – how do you feel about it now?” The response options included: “I wish I’d waited longer before having sex”; “I wish I’d not waited so long”; “It was at about the right time”; “It shouldn’t have happened at all” and “I prefer not to say”. When participants selected “I wish I’d waited longer before having sex” and “It shouldn’t have happened at all”, this was regarded as an indication of regret or disappointment related to their first sex experience.

*Well-being* was measured using the Flourishing Scale for well-being, comprising 8 items for an individual’s self-perceived success in areas such as relationships, self-esteem, purpose, and optimism (Diener et al., 2010). These items describe important aspects of human functioning ranging from positive relationships, to feelings of competence, to having meaning and purpose in life (Diener et al., 2010). The scores range from 8 (being the lowest level of well-being) to 56 (being the highest level of well-being) (Cronbach’s Alpha: 0.78). Well-being is an outcome variable in the analysis referring to whether early sexual debut has an effect on emotional well-being.

### Study procedures

Surveys were conducted in private spaces, inside or outside households, where the participants felt comfortable. Fieldworkers administered surveys electronically using tablets. Sensitive questions regarding sexual behavior were self-administered by AGYW in privacy. Surveys were conducted in participants’ preferred languages (English, Afrikaans, isiXhosa, isiZulu, Sesotho, Sepedi, Setswana, Xitsonga). Participants who disclosed abuse or suicidal tendencies/thoughts to the fieldworker were referred to a social worker contracted to the study. Participants were reimbursed with a gift voucher to the value of R75 (∼USD4) for time spent participating. The study was approved by the South African Medical Research Council Ethics Committee. The study was also reviewed in accordance with the U.S. Centers for Disease Control and Prevention (CDC) human research protection procedures and determined to be research, but CDC investigators did not interact with human subjects or have access to identifiable data for research purposes. Provincial health approval was received from the Department of Health in each province. All participants signed consent forms. For those under 18 years, caregiver consent was obtained before receiving assent from the participant themselves.

### Data analysis

Analysis was restricted to those who reported ever having had penetrative sex (*N* = 3009). Data were analyzed using STATA/SE 14.2 (StataCorp, 2015). Survey-based analytical techniques were used, specifying SALs as the primary sampling unit, districts as our strata, the number of SAL in each district as the finite-population correction, and sample weights. Descriptive summary statistics were used to describe participant characteristics for those who reported to have had sex and the circumstances of their first sex. Hypothesis testing was performed using Chi-square tests to compare timing of sexual debut (outcome variable) with associated factors (independent variables) including SRH and regret/disappointment. The Wilcoxon rank sum test was used to observe differences in the distribution of well-being scores between early and later sexual debut. The Wilcoxon test did not take into account the survey study design. A linear regression accounting for sampling design and weights, was performed to assess the extent to which there is a relationship between well-being at the time of the survey (outcome variable) and early sexual debut (explanatory variable), adjusting for age group, relationship status, and SES group.

## Results

### Socio-demographic characteristics

A total of 3009 (69.2%) AGYW participated in the survey indicated that they had ever had penetrative sex. The median age was 20 years (interquartile range (IQR): 18–22) and the majority were in the 20–24 age group (Table 1). Majority of AGYW reported to have been dating someone at the time of the survey (75.1%). Less than half (44.0%) of participants reported that they were in school at the time of the survey. Orphanhood was common in the study population (maternal orphan: 22.4%; paternal orphan: 36.8%; double orphan: 12.0%). Majority of participants (79.7%) were considered to be in the low socio-economic group.

**Table 1.** Socio-demographic characteristics of adolescent girls and young women who participated in a household survey between 2017 and 2018.

Variable	Frequency (%)
Age group	
15–19 years	1300 (43.3%)
20–24 years	1709 (56.7%)
Relationship status	
Single	638 (23.1%)
Dating	2317 (75.6%)
Married	34 (1.1%)
Missing	20 (0.2%)
Currently in school	
Yes	1340 (44.0%)
No	1669 (56.0%)
Orphan status	
Maternal orphan	
Yes	721 (22.4%)
No	2241 (76.1%)
Missing	47 (1.6%)
Paternal orphan	
Yes	1142 (36.8%)
No	1833 (62.1%)
Missing	34 (1.1%)
Double orphan	
Yes	401 (12.0%)
No	2560 (86.4%)
Missing	48 (1.6%)
SES* Indicator	
Low socio-economic group	2460 (79.7%)
High socio-economic group	549 (20.3%)

*SES: socio-economic status.

### Description of participants’ sexual debut, subsequent feelings about sexual debut, and well-being

The median age of sexual debut was 17 years (IQR: 16–18). Table 2 shows that 259 (8.9%) AGYW had an early sexual debut, with slightly more AGYW in the younger age group reporting an early sexual debut (12.1 vs. 6.4%). The majority of 15–19 year olds had their first sex with a boyfriend who was the same age as themselves (66.0%). The majority of both 15–19 year olds (68.5%) and 20–24 year olds (72.4%) reported their first sexual experience to be something they wanted, while fewer reported it to be something they did not want, that they had been forced against their will or raped. Over half of AGYW in both age groups reported condom use at sexual debut. Modern contraceptive use at sexual debut was reported by 23.5% of 15–19 year olds and 22.4% of 20–24 year olds. Dual protection at sexual debut was reported by 15.3% of 15–19 year olds and 15.0% of 20–24 year olds. When asked about their feelings regarding their first sex, the majority of AGYW in both age groups wished they had waited longer to have sex (15–19 years: 52.8%; 20–24 years: 51.0%). The majority of AGYW reported high levels of well-being regardless of age category.

**Table 2.** Description of participants’ sexual debut, subsequent feelings about sexual debut, and well-being among 3009 adolescent girls and young women aged 15–24 years who participated in a household survey between 2017 and 2018.

Variable	15–19 Years at time of survey (*N* = 1300)	20–24 Years at time of survey (*N* = 1709)	Total (*N* = 3009)
Sexual debut			
Early sexual debut	153 (12.1%)	106 (6.4%)	259 (8.9%)
Later sexual debut	1147 (87.9%)	1603 (93.6%)	2750 (91.2%)
First sexual partner			
Boyfriend (own age)	843 (66.0%)	997 (59.3%)	1840 (62.2%)
Boyfriend (+-5yrs older)	314 (23.1%)	530 (29.8%)	844 (26.9%)
Boyfriend (+-10yrs older)	44 (3.0%)	79 (4.6%)	123 (3.9%)
Blesser	5 (0.5%)	4 (0.2%)	9 (0.3%)
Casual partner	18 (1.6%)	19 (1.2%)	37 (1.3%)
Stranger	16 (1.2%)	10 (0.7%)	26 (0.9%)
Father	1 (0.1%)	3 (0.2%)	4 (0.1%)
Husband	2 (0.2%)	17 (1.2%)	19 (0.7%)
Teacher	3 (0.2%)	1 (0.1%)	4 (0.1%)
First sexual experience			
Wanted	867 (68.5%)	1217 (72.4%)	2084 (70.7%)
Not wanted	249 (17.9%)	288 (15.8%)	537 (16.7%)
Forced to against my will	23 (1.8%)	36 (2.0%)	59 (1.9%)
Raped	21 (1.6%)	19 (1.1%)	40 (1.3%)
First sex condom use			
Yes	808 (62.5%)	1039 (61.4%)	1847 (61.9%)
Pregnancy prevention			
Modern contraceptive use other than condoms	299 (23.5%)	366 (22.4%)	665 (22.9%)
Dual protection	193 (15.3%)	242 (15.0%)	435 (15.1%)
Subsequent feelings about first sex			
Wish waited longer	688 (52.8%)	882 (51.0%)	1570 (51.8%)
Wish did not wait so long	107 (8.0%)	138 (7.6%)	245 (7.8%)
Was the right time	244 (19.1%)	399 (24.8%)	643 (22.3%)
Shouldn’t have happened at all	221 (16.7%)	238 (13.3%)	459 (14.8%)
Well-being			
Median score	47 (IQR*: 43-50)	47 (IQR*: 43-50)	47 (IQR*: 43-50)

*IQR: interquartile range.

### Comparisons of the first sex experiences of AGYW who had an early sexual debut with those who had a later sexual debut

Table 3 compares the first sex experiences of AGYW who had an early sexual debut with those who had a later sexual debut, stratified by participants’ age at the time of the survey. A smaller fraction of participants who had an early sexual debut had their sexual debut with a lower risk partner than those who had a later sexual debut, in both age groups (*p*-value <0.001). Nine percent (9.4%) of 15–19 year olds and 12.5% of 20–24 year olds who had an early sexual debut had been coerced at their first sex compared to 2.3% of 15–19 year olds and 2.3% of 20–24 year olds with a later sexual debut (*p*-value <0.05).

**Table 3.** Comparison of first sexual experience characteristics, subsequent feelings about sexual debut and well-being by sexual debut status among adolescent girls and young women aged 15–24 years who participated in a household survey between 2017 and 2018.

	15–19 years			20–24 years										
	Early sexual debut			Later sexual debut				Early sexual debut			Later sexual debut			
Response options	*n*	%	95%CI	*n*	%	95%CI	*p* Value	*n*	%	95%CI	*n*	%	95%CI	*p* Value
Lower risk partners	111	72.3	65.3–78.3	1048	91.7	90.3–92.8	0.00*	78	71.9	64.1–78.5	1466	91.4	90.3–92.4	0.00*
Coerced	15	9.4	5.6–15.1	28	2.3	1.8–3.1	0.00*	14	12.5	8.3–18.3	39	2.3	1.8–3.0	0.00*
First sex condom Use	76	49.7	42.9–56.4	732	64.3	61.9–66.6	0.00*	51	49.3	41.8–56.8	988	62.3	60.1–64.4	0.00*
Contraception use	35	22.0	16.6–28.6	264	23.7	21.5–26.1	0.58	23	20.2	14.8–27.0	343	22.6	20.7–24.5	0.49
Dual protection	14	8.6	5.8–12.8	179	16.2	14.4–18.2	0.00*	10	9.9	6.0–15.9	232	15.3	13.7–17.0	0.08
Regret/Disappointment	102	67.0	60.3–73.0	779	67.6	64.9–70.2	0.86	79	71.4	64.0–77.8	1014	62.1	59.8–64.3	0.02*
	Median (IQR**)			Median (IQR**)				Median (IQR**)			Median (IQR**)			
Well–being	47 (IQR**: 20–56)			47 (IQR**: 11–56)			0.04*	46 (IQR**: 30–56)			47 (IQR**: 14–56)			0.09

*0.05 or <0.05.

**IQR: interquartile range.

In both age groups, AGYW who had an early sexual debut had lower prevalence of using a condom at first sex (*p*-value <0.001) compared with those who had a later debut. Among AGYW aged 15–19 years, only 8.6% used dual protection at their first sex compared to 16.2% of those with late sexual debut (*p*-value <0.001).

Nearly three-quarters (71.4%) of AGYW aged 20–24 years who had an early sexual debut had regret/disappointment related to their first sex, while 62.1% of those with late sexual debut had regret or disappointment (*p*-value = 0.02). There was little observable difference in the proportions experiencing regret or disappointment among AGYW aged 15–19. With regards to well-being among 15–19-year old AGYWs, a difference was not observed in the median scores of well-being between early and later sexual debut (Median: 47 (IQR: 11–56). Among those aged 20–24, there was a slight difference in well-being median scores with those with an early sexual debut scoring slightly lower (Median: 46 (IQR: 30–56)) than those with a later sexual debut (Median: 47 (IQR: 14–56)). These differences were not significant (*p*-value = 0.09). While there were no significant differences in *medians* of wellbeing scores, a Wilcoxon rank sum test showed that there was a statistically significant difference in the distributions between the two groups among 15–19-year olds. The probability of a 15–19-year old with a later sexual debut having a well-being score that is higher than the well-being score in early sexual debut group is 55.8%.

### The relationship between early sexual debut and well-being

A linear regression model was constructed to predict the relationship between early sexual debut and well-being. Those with a later sexual debut had on average a well-being score 1.14 points higher than those with an early sexual debut (95% CI: 0.39 − 1.89), adjusting for age group, relationship status and SES group. Participants aged 20–24 years old had 0.14 points higher well-being than those aged 15–19 years (95% CI:−0.23 − 0.51), but not statistically significant. AGYW who were dating and married had 0.52 (95% CI: 0.02 − 1.02) and 1.90 (95% CI: 0.29 − 3.52) points higher well-being than those who were single. While, those in the high socio-economic group had 1.84 points higher well-being than those in the low socio-economic group (95% CI:1.41 − 2.27) (Table 4).

**Table 4.** Relationship between sexual debut and well-being at the time of the survey, adjusting for age, relationship status, and SES among adolescent girls and young women aged 15–24 years who participated in a household survey between 2017 and 2018.

*Independent variable	β-coefficients (95% CI)
Sexual debut	
Early sexual debut	(REF)
Later sexual debut	1.14 (0.39 − 1.89)
Age group	
15–19-year olds	(REF)
20–24-year olds	0.14 (-0.23–0.51)
Relationship status	
Single	(REF)
Dating	0.52 (0.02–1.02)
Married	1.90 (0.29–3.52)
SES** indicator	
Low socio-economic group	(REF)
High socio-economic group	1.84 (1.41–2.27)

*First category is a reference.

**SES: socio-economic status.

## Discussion

This study has shown that a larger proportion of AGYW in the younger aged group (15–19 years) had an early sexual debut, compared to those in the older age group (20–24 years) as expected. AGYW in both age groups who had an early sexual debut were more likely to have been coerced at first sex, less likely to have had first sex with a lower risk partner, and less likely to have used a condom at first sex, compared with those who had a later sexual debut. AGYW aged 15–19 years who had an early sexual debut were less likely to have used dual protection than those of the same age who had a later debut. The associations we found of early sexual debut and age-disparate sexual partnerships concur with a previous South African study which found that for each additional year that a young woman's first partner was older than she was, the likelihood that she had had an early sexual debut increased significantly (adjusted prevalence ratio, 1.1) (Pettifor et al., 2009).

This study has shown that young women aged 20–24 years who had an early sexual debut were more likely to report regretting their first sex, and to have a lower well-being score, compared with young women of the same age who had a later sexual debut. Research shows that people reflect on their past experiences during the course of their life span, (Staudinger, 2001) and often regret past choices, especially when misaligned with current needs, values or desires (Wrosch et al., 2005). In addition, the emotion or regret is associated with feelings of self-blame, imagining that one’s current situation would be different had one acted differently (Oswalt et al., 2005). One would imagine that with South Africa having high gender inequality balances, the gender norms and expectations placed on young women about shame in engaging in sex could possibly have influenced the regret felt after engaging in sex at an early age.

Although the majority of AGYW in our study reported their first sexual partner to have been with someone the same age as themselves (62.2%), when comparing early sexual debut versus later sexual debut, it was found that AGYW who had an early sexual debut were less likely to have had their sexual debut with someone who was the same age, 5 years older, or a husband. This meant that they were more likely to have a partner who was 10 years older, a blesser, casual partner, stranger, father or teacher; putting the AGYW at risk for HIV (Luke, 2005; Mercer et al., 2006) and could indicate the AGYW was persuaded or coerced by these first partners at an early age.

The majority of AGYW in our study reported that their first sex was “something they wanted”, however, when asked about subsequent feelings about their first sex, the majority reported that they wished they had waited longer to have sex. This suggests even though they wanted their first sex at the time it happened, when reflecting on it later, they felt they should have waited longer before having sex. When comparing those who had an early sexual debut and those who had a later debut, we found AGYW aged 20–24 years with an early sexual debut were significantly more likely to have regret/disappointment related to their first sex, while there were no significant differences found among 15–19 year olds. Perhaps this alludes to older participants having had more time to think about their sexual debut and to reassess their sexual debut in the light of current values and preferences. No other studies were found comparing the prevalence of regret/disappointment among those who had an early and later sexual debut, however, a significant amount of research has looked at anticipated regret and how it motivates people’s actions (Brewer et al., 2016). Brewer’s findings from a meta-analysis suggest that anticipated regret is an emotion that influences decision-making and may therefore be different from other anticipated negative emotions. Regret management theory suggests that people act to reduce the regret they experience and expect to experience from blaming themselves (Pieters & Zeelenberg, 2007). Awareness of the regret that may follow early sexual debut could facilitate more informed decisions among young women about sexual debut.

Findings regarding the relationship between well-being and sexual debut could not be compared to other studies because, to our knowledge, this is the first study to have made this comparison globally. Previous studies have however been found to support the asscociation between being in a romantic relationship and having a higher well-being regardless of the quality of the relationship (Girme et al., 2016; Kim & McKenry, 2002; Waite, 2002). A recent publication looked into the concept of sexual well-being and argued∥that it is a meaningful indicator of overall well-being (Mitchell et al., 2021) and is crucial in addressing sexual health inequalities in the field of public health.

### Limitations

A limitation is that we did not make a distinction between vaginal and anal penetrative sex, to understand differences in regret, or conceptualisations of ‘virginity’ and sexual debut, associated with different sex acts (Duby, 2019). The classification of AGYW’s sexual partners as “lower risk partners” and “higher risk partners” might not reflect the actual risk of individual partners and therefore the validity of this classification is unknown. The classification of sexual partners into higher and lower risk groups does not exclude risk totally from any particular type of partner, even those grouped as lower risk in this paper. Despite putting in place procedures for self-completion of questions about sexuality, underreporting and social desirability bias may have affected findings. This study was unable to determine why participants felt regret or disappointment about their first sex.

## Conclusions

The findings of this study have important implications for sexual and reproductive health during adolescence and adulthood and can help highlight priority areas of focus for interventions to delay sexual debut. Interventions aiming at delaying sexual debut could include a focus on HIV awareness, sexual attitudes and peer pressure which have been found in previous studies to be significantly associated with early sexual debut among young men (Chirinda et al., 2012). Our findings revealing the extent to which first sex was coerced in cases of early sexual debut clearly demonstrate the importance of interventions which combat violence against women and children and promote gender-transformative social norms. Interventions conducted in South Africa such as the Stepping Stones and Creating Futures combined intervention (Jewkes et al., 2014) which aimed to reduce gender-based violence and HIV risk among young men and women. This combined intervention proved to be successful in reducing men’s controlling behaviors toward partners as well as reduced sexual and physical IPV experienced by women. Couple-based programmes that focus on developing healthy relationships where partners feel respected and able to trust each other, spend time together engaging in activities, and use good communication and problem-solving skills might be useful to prevent IPV and HIV (Belus et al., 2018). The PREPARE project, a school-based HIV and IPV prevention programme aimed to delay sexual debut, increase condom use and decrease IPV (Mathews et al., 2016). They found the intervention arm had significantly better condom and HIV/AIDS knowledge and lower rates of IPV compared to the control arm. Programs to delay sexual debut have also shown promising results through the use of HIV and sexuality education that meets established standards (Aarons et al., 2000; Kirby et al., 2006). Efforts should be made to further increase awareness among AGYW relating to the potential adverse consequences of early sexual debut (including regret and compromised well-being), and interventions should empower young people to make informed decisions around sexual debut, recognizing that they do not always have the power to implement such decisions. Further research into the consequences of regret in adulthood maybe useful.

## Data Availability

Dataset can be made available on special request.
